# “Ethics or Justice”—Which?

**Published:** 1906-06

**Authors:** 


### "Ethics or Justice"—Which?

Consistency, rare gem, how beautiful thou art.

"Look on this picture— and on this."

| THE "ETHICAL" MEDICINE MAKER. | THE "ETHIC-SHOUTING" PHYSICIAN. |
|---|---|
| The ethical medicine maker conducts exhaustive and most expensive experiments to learn the value of a product. Unless he gives his successful produce some trade-mark name, he has absolutely no protection whatever. | The ethic shouting physician writes a book, "The Practice of Medicine." It is copyrighted (thus protecting the fruit of his brain, and the publishers pay him a royalty (thus properly and justly rewarding his labors). He simply can not lose. |
| The unfavorable experiments of the ethical medicine maker cost him thousands and thousands of dollars. His time, his experience and his money have been freely | The unfavorable experiments and the unfortunate mistakes of the physician are usually known to himself only. His bill for services is sent just the same and is |

used; his reputation, his conscience, his business judgment will not permit him to present a product which is faulty. The unworthy product is consigned to the garbage pile — no one reimburses him for the outlay connected with his unprofitable investigation. The money is "gone, but not forgotten."

paid by the executors (if there be anything left).

---

The ethical medicine maker confines his operations to ethical channels. If he gives his product an arbitrary name (to distinguish it, thus guaranteeing that the patient gets *the* product, not any old thing) he is guilty of a crime. If one of his clinical reports or pamphlets (intended for medical practitioners only) chances to get into the hands of a layman, the medicine maker has committed an unpardonable sin and his product is tabooed by certain self-appointed "ethical" judges, the most pronounced of all being the very ones who crave fame before the public eye, and use for its accomplishment the very methods which they would damn the medicine maker for employing, which would, in fact, put him to shame. The medicine maker must pay for any advertising he secures.

The "ethical physician is quoted in the secular press, writes books of fiction or otherwise for sale among the laity, and at least one prominent physician permitted his name to be heralded in a leading New York daily paper in an advertisement which announced the forthcoming appearance of an article from his pen, which was to record the progress in medicine during the century just closed, thus the politic physician is paid handsomely for getting himself advertised broadcast throughout the land. That same physician has received enormous compensation in royalty on the sale of his copyrighted books—thus securing to himself the protection which he would withhold from the ethical medicine maker.

---

The medicine maker is asked to furnish to a hospital a sufficient quantity of his product to make a comparative and conclusive test. If the test shows the product to be of less value than the recognized remedies already in use, his product is discarded by the physician. If it proves superior to the old remedies, the physician, having kept minute data regarding the tests, feels the facts to be of value to his colleagues, reads a paper before some society relating his clinical experience, *stating exactly the product* (or the name of its maker) through which his results were secured.

He sends his paper to a medical journal.

The "ethical" medical editor blue pencils the name of the product or the name of its maker (unless it chances to be European, which, of course, makes a profound difference). He thus destroys the force of the report, places words in the author's mouth which he did not utter, does an injustice to his readers by withholding the actual clinical facts which made the report of value in the first place, and is guilty of ingratitude and injustice toward the medicine maker, who, working honestly and for an honest end is thus deprived of the credit which honestly belongs to him.

Please remember that the journal over which this same editor presides is copyrighted. If another journal quotes from its pages without giving proper credit the charge of plagiarism and literary theft is promptly made.

Some physicians will not even try a proprietary article until having exhausted the usual list of remedies. The proprietary article is frequently not used until the patient is almost in a hopeless condition, thus the proprietary article must win its spurs under conditions which are manifestly unfair.

The "ethic shouting" physician writes a prescription, but, instead of specifying the proprietary article which benefited his patient, he states the names of the ingredients thereof.

The prescription is thus filled extemporaneously, while the proper product may have taken three or four months to properly compound.

Is that fairness to the manufacturing chemist or justice to the patient?

